# Pharmacophylogenetic study of *Scutellaria baicalensis* and its substitute medicinal species based on the chloroplast genomics, metabolomics, and active ingredient

**DOI:** 10.3389/fpls.2022.951824

**Published:** 2022-08-17

**Authors:** Jie Shen, Pei Li, Yue Wang, Kailing Yang, Yue Li, Hui Yao, Qiang Wang, Peigen Xiao, Chunnian He

**Affiliations:** ^1^Key Laboratory of Bioactive Substances and Resources Utilization of Chinese Herbal Medicine, Ministry of Education, Institute of Medicinal Plant Development, Chinese Academy of Medical Sciences, Peking Union Medical College, Beijing, China; ^2^School of Medical Laboratory, Weifang Medical University, Weifang, China; ^3^State Key Laboratory of Systematic and Evolutionary Botany, Institute of Botany, Chinese Academy of Sciences, Beijing, China

**Keywords:** *Scutellaria baicalensis*, pharmacophylogeny, substitute medicinal species, chloroplast genomics, metabolomics

## Abstract

The genetic relationships among the species in *Scutellaria* genus remain unclear because of the variation in the number of species and complex trait. The usage of *S. baicalensis* and its four substitute medicinal species (*S. amoena*, *S. hypericifolia*, *S. likiangensis*, and *S. viscidula*) in traditional medicines make their specialized metabolism important in China, but interspecific genetic and chemical differences have rarely been reported for these species. In this study, the chloroplast genomes of four substitute species for *S. baicalensis* were assembled, and comparative and phylogenetic analyses were performed with these species and other *Scutellaria* relatives. In addition, metabolomics analyses were performed and the contents of the main active compounds were determined to reveal the interspecific chemical diversity of *S. baicalensis* and its four substitute species. The full lengths of their chloroplast genomes ranged from 151,574 to 151,816 bp with an average GC content of 38.34%, and a total of 113 genes were annotated. In the chloroplast genomes of *S. baicalensis* and its four substitutes, one hypervariable region (*petA*-*psbL*) is proposed as a potential DNA barcode. Phylogenetic analysis showed that the subdivision of the genus *Scutellaria* should be reconsidered. The metabolomics and content determination analyses showed that the four species exhibit a metabolism similar to that of *S. baicalensis* in different parts. Except for the roots of *S. likiangensis*, all parts of the substitute species showed high contents of baicalin. Genetic and chemical analyses of four substitute medicinal species for *S. baicalensis* were performed here for the first time, and their pharmacophylogenetic relationships were further explored, providing a scientific basis for the subsequent development of the medicinal value and resource utilization of *Scutellaria*.

## Introduction

*Scutellaria* is a significant genus in the family Lamiaceae, which includes approximately 360–469 accepted species worldwide (Ranjbar and Mahmoudi, 2017; Safikhani et al., 2018; Zhao et al., 2020; Shen et al., 2021). According to records, 72 species in this genus have been used with a long history as traditional herbal medicines to treat various diseases, such as cancer and inflammatory, hepatic, gastric, neurological, and cardiovascular diseases (Marsh et al., 2014; Grzegorczyk-Karolak et al., 2016; Wang et al., 2018, 2020). *Scutellaria* species are rich in flavonoids and terpenoids, and the main compounds baicalein, baicalin, and wogonin have been evaluated in clinical trials (Shen et al., 2021).

In China, *S. baicalensis* is the most widely studied species in *Scutellaria*. Scutellariae Radix, consisting of the roots of *S. baicalensis*, has been used as a traditional Chinese medicine for thousands of years and is officially recorded in successive editions of the *Chinese Pharmacopoeia* (Wang et al., 2018). According to research, the roots of some closely related species, such as *S. amoena, S. hypericifolia, S. likiangensis*, and *S. viscidula*, are similar in shape to the roots of *S. baicalensis*, and they have abundant natural medicinal resources and have been widely used as substitutes for Scutellariae Radix in China (Shen et al., 2021). These alternatives have been used as herbal folk medicines for a long time and are listed in some local flora and local standards for traditional Chinese medicine. In the current *Sichuan Standard for Traditional Chinese Medicine* (2010), Scutellariae Amoenae Radix (Chuan Huang Qin) originating from the roots of *S. amoena*, *S. hypericifolia*, and *S. tenax* var. *patentipilosa* is used to prevent miscarriage and bleeding. In addition, in the current standard *Quality standard of Chinese and ethnic medicinal materials in Guizhou Province* (2003), the root of *S. amoena* is listed as Scutellariae Amoenae Radix (Xi’nan Huangqin) and described as useful for treating fever, cough, hemoptoe, jaundice, diarrhea, and carbuncles and for preventing miscarriage. Furthermore, the root of *S. viscidula* was listed in the *Drug Standard of Jilin Province* (1977) as Radix Scutellariae Viscidula and described as useful for treating cough, jaundice, diarrhea, and swelling and pain of the eye and for preventing miscarriage. Despite their important medicinal value, the genetic and chemical diversity of these substitutes for *S. baicalensis* remains unclear.

According to the Flora of China (Wu and Li, 1977), S. baicalensis and its substitute species (S. amoena, S. hypericifolia, S. likiangensis, and S. viscidula) all belong to the Scutellaria subgen. Scutellaria sect. Stachymacris subsect. Angustifoliae clade, which has high similarity in terms of morphology and root shape. To date, only one study has used the plastid molecular marker trnL-F to discriminate between S. baicalensis and its adulterants (*S. amoena* and *S. viscidula*) (Wang et al., 2012). In recent years, chloroplast (cp) genomes have played an essential role in identifying closely related plant species and performing phylogenetic analysis since cp DNA is more discriminating and versatile than nuclear DNA sequences (Cui et al., 2019a; Zhao et al., 2020). The cp is an essential photosynthetic organelle in plants (Daniell et al., 2016). The cp is a uniparentally inherited plastid that contains circular double-stranded DNA (Zhao et al., 2020). A typical angiosperm cp genome has a four-part structure that includes two identical regions in opposite orientations called inverted repeats (IRs), separated by large single-copy (LSC) and small single-copy (SSC) regions (Ruhlman and Jansen, 2014). *Scutellaria* is a very isolated genus with unsatisfactory traditional divisions, and complete cp genomes have been published for only 13 species in the genus (Jiang et al., 2017; Lee and Kim, 2019; Zhao et al., 2020). In addition, little is known regarding the plastome structural variation among S. baicalensis and its substitutes. Thus, sequencing the cp genomes of these four Scutellariae Radix substitute species is beneficial not only for accurately identifying closely related species but also for greatly contributing to medicinal cp genetic development in *Scutellaria*. In addition, until now, among the substitute species for S. baicalensis, only one complete cp genome (S. amoena) of the subsect. Angustifoliae clade has been published. The availability of complete cp genome sequences of S. hypericifolia, S. likiangensis, and S. viscidula will help reveal the evolutionary pattern of the complete cp genome in the subsect. Angustifoliae clade.

Plant secondary metabolites usually provide the ultimate features for distinguishing among similar species (Nguyen et al., 2016). The phytochemical diversity of plant metabolites could provide a basis for our understanding of the evolution and classification of different plant species (Wink, 2015). Currently, metabolomics is considered the most common method for profiling and comparing phytochemical compositions among species (Kushalappa and Gunnaiah, 2013). To date, a large number of chemical components have been identified from the genus *Scutellaria*, and flavonoids and diterpenes have been confirmed to be the two main groups of active constituents in this genus. Based on the pharmacophylogenetic theory, plants with similar therapeutic effects are phylogenetically related and often similar in chemical composition. However, due to the lack of adequate chemical composition and content determination studies, additional evidence is needed to confirm the relationship between chemical diversity and phylogeny in *Scutellaria* (Shen et al., 2021). Previous studies have indicated that the major flavonoids baicalin, baicalein, wogonoside, and wogonin are 4′-deoxyflavones unique to the roots of *S. baicalensis* with a wide range of pharmacological activities, and high concentrations of these compounds have also been detected in many *Scutellaria* plants. However, only two substitutes (*S. amoena* and *S. viscidula*) for *S. baicalensis* have been reported to contain compounds similar to those in *S. baicalensis*, such as baicalein, baicalin, and wogonin (Wang et al., 2012; Ling et al., 2016). Moreover, the chemical profiles of these four substitutes (*S. amoena*, *S. hypericifolia*, *S. likiangensis*, and *S. viscidula*) for *S. baicalensis* remain to be completely explored. Currently, metabolomics is considered the most common method for profiling and comparing phytochemical compositions among species (Kushalappa and Gunnaiah, 2013). Due to the significant differences in chemical composition between the aerial parts and roots of *S. baicalensis* previously reported (Shen et al., 2019), in this study of the four species mentioned above, we divided *S. baicalensis* and its four substitute species into aerial parts and roots for separate metabolomics determination.

In this study, we first sequenced the complete cp genomes of *S. amoena*, *S. hypericifolia*, *S. likiangensis*, and *S. viscidula* by using Illumina sequencing technology. Comparative analyses were conducted to explore the different characteristics of the cp genomes of these four substitutes for *S. baicalensis*. In addition, a phylogenetic tree was constructed to reveal the phylogenetic positions of these medicinal plants in *Scutellaria*. Furthermore, the chemical profiles of metabolites in different plant parts were determined by ultra-performance liquid chromatography/quadrupole time-of-flight mass spectrometry (UPLC-QTOF-MS), and chemometric analyses (PCA, PLS-DA, and HCA) were conducted. In addition, high-performance liquid chromatography (HPLC) analysis was used to determine the 15 main flavonoids in different parts of *S. baicalensis* and its four substitute species. Analyses of phylogenetic relationships and phytochemical diversity among the four substitute species for *S. baicalensis*, in this study, can expand our understanding of the genetics and chemistry of the genus *Scutellaria*.

## Materials and methods

### Plant materials

Fresh whole plants of *S. amoena, S. baicalensis, S. hypericifolia, S. likiangensis*, and *S. viscidula* were collected from the Gucheng District (Lijiang City, Yunnan Province), Baihua Mountain (Beijing), Luoshage (Dali Bai Autonomous Prefecture, Yunnan Province), Wenhai Road (Yulong Naxi Autonomous County, Lijiang City, Yunnan Province), and Wulan Halaga (West Ujimqin, Inner Mongolia Autonomous Region) in China, respectively. Three samples were collected at each site in August 2019 and were divided into two parts: aerial parts and roots. The samples were authenticated by Professor Qiang Wang (State Key Laboratory of Systematic and Evolutionary Botany, Institute of Botany, Chinese Academy of Sciences), and voucher specimens (HQS-01, *S. amoena*; HQS-02, *S. baicalensis*; HQS-03, *S. hypericifolia*; HQS-04, *S. likiangensis*; and HQS-05, *S. viscidula*) were deposited in the pharmacophylogeny research center (Institute of Medicinal Plant Development, Chinese Academy of Medical Sciences, Peking Union Medical College, Beijing, China).

### DNA extraction and sequencing

Fresh leaves were collected and dried using silica gel for DNA extraction. Genomic DNA was extracted using the Plant Genomic DNA Kit (Huayueyang, Beijing, China) according to the instructions. Then, 1% (w/v) agarose gel electrophoresis was used to test DNA integrity, and concentrations were determined using a NanoPhotometer^®^ spectrophotometer (IMPLEN, Munchen, Germany) and a Qubit 2.0 Fluorometer (Life Technologies, Carlsbad, CA, United States). Finally, high-quality DNA was used for library construction and sequencing on any Illumina platform, and 150-bp paired-end reads were generated.

### Chloroplast genome assembly, annotation, and structural analysis

K-values of 21, 55, 85, and 115 were obtained using GetOrganelle software (Jin et al., 2018) to obtain the desired results, and the entire genome of *S. baicalensis* Georgi (NC_027262.1) from the National Center for Biotechnology Information (NCBI) was used as the reference for cp genome assembly.

Chloroplast annotation of the assembled sequences was performed using GeSeq^1^ (Michael et al., 2017). These sequences were compared with the known database, and sequences of the protein-coding gene BLAT and the rRNA/tRNA gene BLAT were identified based on similarity values exceeding 85%. In addition to the default known database in GeSeq, the cp genome annotation process also included the addition of the entire genome of *S. baicalensis* from the NCBI database. Based on the GeSeq annotation results, the software used to create a circular physical map of the cp genome was OGDRAW^2^ (Stephan et al., 2019).

The GC content of the cp genome was analyzed using MEGA-X (Kumar et al., 2018). The MISA Perl script^3^ (Beier et al., 2017) was used to identify simple sequence repeats (SSRs). The definition of microsatellites (unit size/minimum repeats) was (1/10) (2/5) (3/4) (4/3) (5/3) (6/3), and the maximum sequence length between two SSRs was set at 100 (Zhao et al., 2020). REPuter (Kurtz et al., 2001) was used to identify the sizes and locations of repeat sequences, including forwarding, palindromic, reverse, and complement repeats, in the cp genomes. The minimal size for all repeat types was 30 bp, with a Hamming distance of 3 (Zhao et al., 2020). The spliced cp genome sequence file was uploaded to the Tandem Repeats Finder v4.09 website^4^ to identify the tandem repeats in cp DNA with default parameters (Benson, 1999).

### Interspecific comparison and phylogenetic analysis

Thirteen reported complete cp genomes of *Scutellaria* were downloaded from the NCBI database: *S. baicalensis* (MF521633), *S. calcarata* (MN128385), *S. indica* var. *coccinea* (MN047312), *S. insignis* (KT750009), *S. lateriflora* (KY085900), *S. mollifolia* (MN128384), *S. orthocalyx* (MN128383), *S. quadrilobulata* (MN128381), *S. kingiana* (MN128389), *S. altaica* (MN128387), *S. przewalskii* (MN128382), *S. scordifolia* (MT712016), and *S. tsinyunensis* (MT544405). The mVISTA web interface^5^ was used to align and compare the five cp genome [*S. amoena, S. baicalensis* (MF521633), *S. hypericifolia, S. likiangensis, S. viscidula*] sequences (Mayor et al., 2000). Shuffle-LAGAN was selected as the alignment program for detecting the rearrangements and inversions in sequences. Multiple sequence alignment of cp genomes was conducted using Condon Code Aligner V.9.0.1 and MAFFT v.7.471^6^ (Frazer et al., 2004). The divergence among different cp genomes and the identification of mutational hotspots were performed by quantifying nucleotide variability in DnaSP v6.12.03 (Rozas et al., 2017). The window length was set to 600 bp, with a 200-bp step size. IRscope^7^ was used to visualize the gene differences at the boundaries of the junction sites of the 14 cp genomes (Amiryousefi et al., 2018). In this study, in addition to *S. baicalensis* and its four substitute species (*S. amoena*, *S. hypericifolia, S. likiangensis, and S. viscidula*), an additional 12 cp genomes were downloaded from NCBI to construct a cp phylogenetic tree. Maximum likelihood (ML) phylogenetic inference was performed using IQ-TREE software (Nguyen et al., 2015) with 1000 bootstrap replicates based on the TVM+F+R6 nucleotide substitution model to assess branch support. MrModeltest 2.3 (Nylander, 2004) was applied to calculate the best-fitting model based on Akaike’s information criterion. Bayesian inference (BI) phylogenetic trees were analyzed in MrBayes version v3.2.7 (Ronquist and Huelsenbeck, 2003) based on the complete cp genome tree.

### Utra-performance liquid chromatography/quadrupole time-of-flight mass spectrometry analysis of specialized metabolites

Dividing each whole plant into the aerial part and root part, all dried samples of the two parts were crushed and screened through a 40-mesh sieve. Samples (25 mg) were macerated with MeOH (25 ml) and placed in an ultrasonic water bath for 30 min. After cooling to room temperature and complementing the reduced volume, each extract was filtered through a 0.25-μm membrane filter, and 1 μL extracts were injected for each analysis. Three biological replicates of the samples were completed using different individuals of the same species.

The UPLC-QTOF-MS analysis was performed on a Waters Acquity Ultra High-Performance LC system (Waters Co., Milford, MA, United States) coupled with a Waters Xevo G2-XS TOF mass spectrometer (Waters Co., Milford, MA, United States) equipped with an electrospray ionization interface. Chromatographic separations were conducted on a Waters Acquity BEH C18 column (2.1 mm × 100 mm, 1.7 μm). The mobile phase was comprised of acidified water with 0.1% formic acid (A) and MeOH (B). The column temperature was maintained at 35°C. The elution program was as follows: 0–3 min: 10–35% B; 3–9 min: 35–70% B; 9–12 min: 70–100% B; and 12–14 min: 100% B. The flow rate was 0.3 mL/min, and the photodiode array detector scanning range was from 200 to 400 nm. The negative mode was used for ionizing the metabolites, and the mass spectra were scanned from 1,000 to 1,200 m/z. The parameters were set as follows: the cone voltage and capillary voltage were set at 40 V and 2.4 kV, respectively. The source temperature and the desolvation temperature were 100°C and 350°C, respectively. The desolvation gas and cone gas rates were 900 and 50 L/h, respectively. Leucine-enkephalin was used as the lock mass in the analyses (negative ion mode: [M-H] ^–^ = 554.2615).

### High-performance liquid chromatography analysis of chemical components in the five *Scutellaria* species

The aerial parts and root samples were all processed according to a previously reported method (Shen et al., 2019). These samples were also analyzed following our previously established protocol (Shen et al., 2019). Three biological replicates of the samples were completed using different individuals of the same species.

### Data processing and analysis

Progenesis QI 2.3 software (Waters, Milford, MA, United States) was used to process the centroid MS*^E^* raw data from UPLC-QTOF-MS, including import data, alignment review, experimental design setup, peak selection, deconvolution, and compound identification. The MetaboAnalyst 5.0^8^ webserver was used for multivariate statistical analyses, including principal component analysis (PCA) and partial least squares discriminant analysis (PLS-DA) in the Pareto scaling mode, hierarchical clustering analysis (HCA) with Euclidean distances, and heatmap analysis.

## Results

### Assembly of chloroplast genomes and comparative analysis of the four substitute medicinal species for *Scutellaria baicalensis*

**FIGURE 1:**
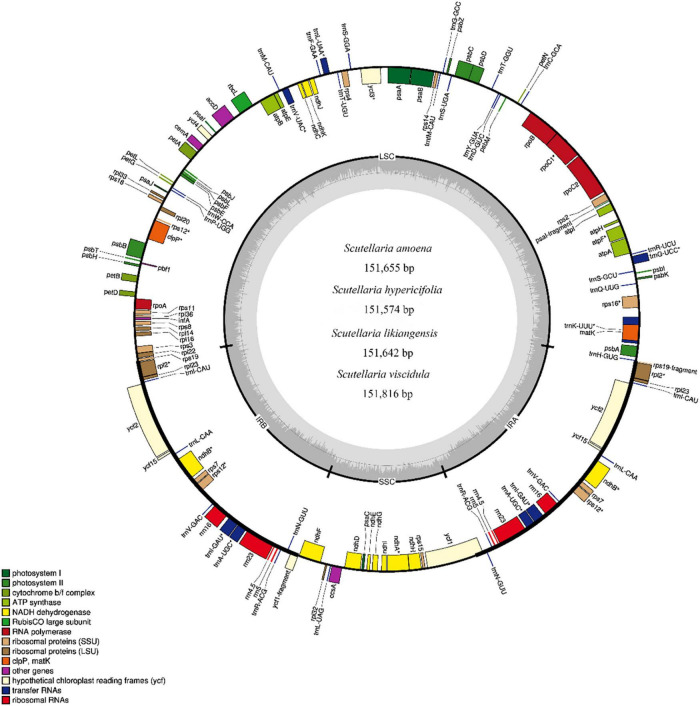
Gene map of the complete cp genomes of *S. amoena*, *S. hypericifolia*, *S. likiangensis*, and *S. viscidula*. Only one map is shown here due to the negligible differences among the four species. Genes inside the circle are transcribed clockwise, while genes outside the circle are transcribed counterclockwise. The different colors indicate different functional groups of genes. Dark gray areas inside the circle indicate the GC content, while light gray areas indicate the AT content of the genome.

Using Illumina HiSeq/MiSeq sequencing platforms, we obtained 3137–3953 M (Illumina raw reads) for four substitute medicinal species of *S. baicalensis* (*S. amoena*, *S. hypericifolia*, *S. likiangensis*, and *S. viscidula*), and 3127–3838 M reads were finally assembled to generate complete cp genomes. The complete cp genomes of the four species were 151,574–151,816 bp in total size (Supplementary Table S1). The lengths were similar in *S. amoena* and *S. likiangensis*. *S. viscidula* was the longest, and *S. hypericifolia* was the shortest. The cp genomes all exhibited the typical quadripartite structure with a large single-copy region (LSC) (83,810–83,995 bp), a small single-copy region (SSC) (17,321–17,326 bp), and a pair of inverted repeats (IRs) (25,253–25,265 bp) (Supplementary Table S1 and Figure 1). The sizes of the SSC and IR regions were identical among *S. amoena*, *S. hypericifolia*, and *S. likiangensis* but differed in *S. viscidula*. The average GC content of the genomes was 38.34%, which is similar to values in other *Scutellaria* species (Lukas and Novak, 2013; Zhao et al., 2020). The GC content was the highest (43.61–43.64%) in the IR regions, the lowest (32.68–32.72%) in the SSC region, and was in the range of 36.34–36.36% in the LSC region (Supplementary Table S1).

In four *Scutellaria* species, a total of 113 genes were identified, including 80 protein-coding genes, 29 tRNA genes, and 4 rRNA genes. Of these, 18 genes were duplicated in the IR regions, including seven protein-coding genes (*rps12, rps7, ndhB, ycf15, ycf2, rpl23*, and *rpl2*), seven tRNAs (*trnN-GUU, trnR-ACG, trnA-UGC, trnI-GAU, trnV-GAC, trnL-CAA*, and *trnI-CAU*), and four rRNAs (*rrn5, rrn4.5, rrn23*, and *rrn16*) (Supplementary Table S2). There were five SSR types (mono-, di-, tri-, tetra-, and pentanucleotides) in these four *Scutellaria* species (Supplementary Figure S1) and 40, 43, 40, and 43 repeat sequences in the *S. amoena*, *S. hypericifolia, S. likiangensis*, and *S. viscidula* cp genomes, respectively. The number of mononucleotide repeats was the highest, accounting for 58.1% of the total number of SSRs, and the number of pentanucleotide repeats was the lowest, accounting for 3.73%. On average, there were approximately 22 tandem repeats, 13 forward repeats, and 17 palindrome repeats. The highest total number of repetitive sequences was found in *S. viscidula*, with 61 duplicate sequences, and the lowest total was found in *S. hypericifolia*, with 50 repetitive sequences (Supplementary Figure S1).

Interspecific comparisons of sequence identity among the cp genomes of *S. baicalensis* and its four substitute medicinal species were conducted with mVISTA, and the annotated *S. baicalensis* sequence was used as a reference. The mVISTA results indicated that the cp genomes of the species of *Scutellaria* all showed a high degree of conservation (Supplementary Figure S2). The variation was more pronounced in the single copy region than in the IR region, and the protein-coding region was more conserved in terms of sequence than the non-coding region. In these five cp genomes of *Scutellaria* species, the non-coding region was more variable than the coding region, and the LSC and SSC regions were more variable than the IR region. Based on the mVISTA results, 6 highly variable regions were found in four species of *Scutellaria*: *trnK* (*UUU*)*-rps16, rps16-trnQ* (*UUG*), *atpH-atpl*, *petN-psbM, trnT* (*GGU*)*-psbD*, and *petA-psbJ* (Supplementary Figure S2). The average nucleotide variability (PI) of the cp genomes of the four *Scutellaria* species was 0.00136 (Supplementary Figure S3), and mutation hotspots with high PI values (>0.01) were distributed in the LSC region with a maximum PI value of 0.0155 (*petA*-*psbL*). The mutational hotspots within these *Scutellaria* species were commonly located in the LSC and SSC regions, as well as the IR region with PI values lower than 0.002. The development and utilization of these highly variable sites are beneficial to phylogenetic research on the subspecies and related genera of *Scutellaria* (Supplementary Figure S3).

The expansion and contraction of IR regions in the cp genomes of these five *Scutellaria* species and 12 other species of *Scutellaria* were compared (Supplementary Figure S4). In all species, the IRa/LSC junction was located within the *rps19* gene. The *rps19* gene had 41–46 bp projections into the IRa region, resulting in the presence of a portion of the *rps19* gene (*rps19* fragment) in the IRb region. The *ycf1* gene straddles the IRb/SSC boundary, but only a small fraction of the gene (771–783 bp) is located in the IRb region, and the pseudogene *ycf1* was detected in the IRa region. The length of *trnH* from the IRa/LSC border was 0 bp.

### Phylogenetic analysis based on chloroplast genomes

**FIGURE 2:**
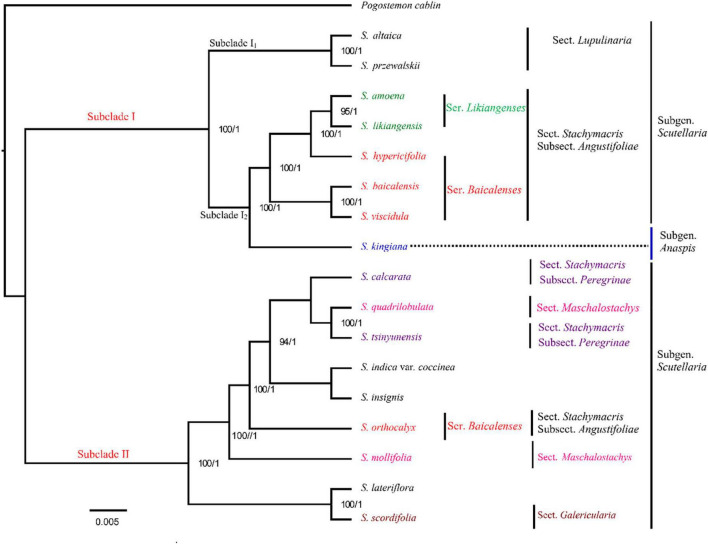
Phylogenetic relationships of the 17 species by maximum likelihood (ML) and Bayesian inference (BI) analyses (*Pogostemon cablin* as an outgroup) based on alignments of complete chloroplast genomes. The numbers above nodes are supported values with ML bootstrap values on the left and Bayesian posterior probability (PP) values on the right.

In this study, based on complete cp genome sequences, 17 species (including the four *Scutellaria* cp genomes newly sequenced in this study) from *Scutellaria* were used for the construction of an ML tree and BI trees (with *Pogostemon cablin* as an outgroup, Figure 2 and Supplementary Figure S5). All species of *Scutellaria* were recovered in two subclades (ML/BS 100, BI/PP 1) (Figure 2). Subclade I (100, 1) comprised eight species. Subclade I is divided into Subclade I_1_ and Subclade I_2_, with 100% support, and these clustered species all have well-developed roots in their morphology. Subclade I_1_ contains two species, *S. altaica* and *S. przewalskii*, both of which are perennial subshrubs with developed woody rhizomes and belong to Subg. Scutellaria Sect. Lupulinaria in the *Flora of China* (Wu and Li, 1977). Subclade I_2_ consists of six species *S. amoena, S. likiangensis, S. hypericifolia, S. baicalensis, S. viscidula*, and *S. kingian*a. In the maximum likelihood tree constructed based on the cp genome, *S. kingiana* was found to have high support (100, 1) for clustering these five species into one, indicating a close affinity. Moreover, *S. kingiana* is a dwarf perennial herb with creeping woody developed rhizomes and belongs to Subgen. Anaspis in the *Flora of China* (Wu and Li, 1977). The species of *S. amoena*, *S. baicalensis*, *S. hypericifolia*, *S. likiangensis*, and *S. viscidula* are clustered together and are all perennial herbs with developed fleshy rhizomes and belong to *Scutellaria* subgen. *Scutellaria* sect. *Stachymacris* subsect. *Angustifoliae* clade in the *flora of China* (Wu and Li, 1977; Lou and Qin, 1995; He et al., 2012). In addition, *S. viscidula* and *S. baicalensis* clustered in one clade with high support (BS value 100), which indicated that they are more closely related. *Scutellaria amoena, S. likiangensis*, and *S. hypericifolia* were clustered in one clade with high support (100,1). Moreover, as substitutes for *S. baicalensis*, the roots of *S. amoena*, *S. likiangensis*, *S. hypericifolia*, and *S. viscidula* are yellow and well developed (Lou and Qin, 1995; Li et al., 2003; He et al., 2012). Subclade II (100, 1) consists of nine species: *S. calcarata, S. lateriflora, S. indica* var. *coccinea, S. insignis, S. mollifolia, S. orthocalyx, S. quadrilobulata, S. tsinyunensis*, and *S. scordifolia* (Figure 2). In Subclade II, all cp sequences from the same clade had high support, and as in Subclade I, the positions of these nine species in phylogenetic trees are also different from the traditional Chinese plant classification system in *Flora of China*. In addition, according to the morphological description of the *Flora of China* (Wu and Li, 1977), most of them are perennial herbs and have no well-developed roots (Figure 2). Therefore, not only the taxonomic positions of the subgenera but also the taxonomic positions of sections and series need further confirmation in the genus *Scutellaria*.

### Specialized metabolites in the *Scutellaria* species

The UPLC-QTOF-MS analyses of the aerial parts and roots of *S. baicalensis* and its four substitute medicinal species (*S. amoena*, *S. hypericifolia*, *S. likiangensis*, and *S. viscidula*) were performed in negative ion mode using electrospray ionization (ESI^–^). Progenesis QI is the most commonly used and reliable software for the online identification of metabolomics data (Gu et al., 2019; Wang et al., 2019). In this study, the chemical composition of these five species was tentatively identified by an online search of an in-house library of the genus *Scutellaria* and a Progenesis MetaScope internal repository through Progenesis QI software and by comparison with literature and standards, as shown in Table 1. Progenesis QI was used with m/z and typical fragment ions through comparison with candidate compounds from various databases. For example, in the online identification by Progenesis QI, baicalin was identified based on molecular weight, adduct ion (m/z 467.0583, M+Na-2H), and typical fragment ion (m/z 269.0431, C_15_H_9_O_5_^–^). The compound apigenin was identified by two adduct ions, M-H (C_15_H_10_O_5_^–^, m/z 269.0699) and 2M-H (C_30_H_18_O_10_^–^, m/z 539.0989), and the fragment ion was identified as C_7_H_3_O_4_^–^ (m/z 151.0039). We further demonstrated the reliability of Progenesis QI identification with reference substances. In the negative ion mode, most of the compounds exhibited deprotonation. The most common compounds were -H_2_O and -CO_2_. Compounds containing carboxylic acid groups commonly lost CO_2_ by negative ionization. Flavonoid glycosides were mainly identified by the loss of character classes of molecules, including the loss of CO and the loss of molecules, by cleavage through the RDA reaction. The loss of sugars such as rhamnose, arabinose, and glucuronide mainly occurred in flavonoid glycosidic compounds. However, similar to other plant species, the species of *Scutellaria* also have specific metabolites, which may contain many isomeric compounds. Given their similar or identical molecular weights, mass spectrometry fragmentation behavior, and minor differences in retention times, it remains challenging to resolve these structural isomers using only mass spectrometry-based metabolomics approaches. Therefore, when identifying compounds by searching for isomers online with Progenesis QI, this study identified compounds based on their polarity, ion fragmentation patterns, and UV absorption wavelengths and referred to literature reports for further confirmation. For example, compounds with retention times of 8.52 min, M-H^–^ (C_18_H_15_O_7_^–^) ions, and 343.0809 m/z were tentatively identified as nevadensin (Liang et al., 2018). The major fragment ions of nevadensin were 241.0496 (M-2CH_2_O- CH_2_-CO), 313.0352 (M-CH_3_), and 328.0572 (M-H-CH_3_). All compounds were identified by the same method. Finally, a total of 75 compounds were putatively identified in negative ion mode, 16 of which were identified by Progenesis QI in agreement with the results of the standards comparison. These compounds included 1 aromatic aldehyde, 2 phenylethanol glycosides, 4 pyrone glycosides, 9 diterpenoids, 55 flavonoids, and 4 other compounds.

**TABLE 1:** Compounds putatively identified from the *Scutellaria* species.

No.	*t*_*R*_ (Min)-m/z (n)	Compound type	Identification	Adduct ion	Molecular formula	UV λ max (nm)	δ (ppm)	[M-H]^–^ measured	[M-H]^–^ predicted	Key MS*^E^* fragment ions (Da)
1	2.16_303.0720m/z	Pyrone glycoside	Scusalvioside A	M-H	C_12_H_16_O_9_	290	–0.375	303.0720	303.0716	174.9530
2	2.52_462.1711n	Pyrone glycoside	2-(3,4-Dihydroxyphenyl) ethyl 3*-O*-(6-Deoxy-α-L-mannopyranosyl)-β-D-glucopyranosi	M-H, M+Cl	C_20_H_30_O_12_	289	–5.7623	461.1638	461.1659	341.0840, 447.1124
3	2.66_465.1020m/z	Pyrone glycoside	Scusalvioside B	M-H	C_21_H_22_O_12_	289	–3.9333	465.102	465.1033	359.0962, 403.1203
4	2.76_300.1187n	Phenylethanoid glycosides	Salidroside	M-H, M+FA-H	C_14_H_20_O_7_	279	–7.2744	345.117	345.1186	299.1122, 174.9557
5	3.05_464.0940n	Flavonol	5,7,2′6′-Tetrahydroxyflavonol-2′*-O*-D*-*glucopyranside	M-H, 2M-H	C_21_H_20_O_12_	278	–3.1591	463.0867	463.0877	287.0537
6	3.25_325.0911m/z	Others	(*E*)-3-[2-[(2*S*,3*R*,4*S*,5*S*,6*R*)-3,4,5-trihydroxy-6-(hydroxymethyl) oxan-2-yl] oxyphenyl] prop-2-enoic acid	M-H	C_15_H_18_O_8_	272	–5.3478	325.0911	325.0923	163.0389, 145.0300, 119.0491
7	3.29_465.1021m/z	Flavononol	Amoenin D	M-H	C_21_H_22_O_12_	289	–3.7262	465.1021	465.1033	347.0784, 285.0387
8	3.39_303.0501m/z	Flavonoid	(2*R*,3*R*)-3,5,7,2′6′-pentahydroxyflavanone	M-H	C_15_H_12_O_7_	288	–3.0489	303.0501	303.0505	285.0378, 259.0575, 77.0169
9	3.54_449.1081m/z	Flavonoid	2,2′-Dihydroxy-3,4,5,6′-tetramethoxy-4′5′-Methylenedioxychalcone	M-H	C_21_H_22_O_11_	281	–1.9534	449.1081	449.1084	287.0534
10	3.61_121.0285m/z	Aromatic aldehydes	2-Hydroxybenzaldehyde*	M-H	C_7_H_6_O_2_	284	–8.0915	121.0285	121.0290	122.0368
11	3.65_476.1875n	Phenylethanoid glycosides	Darendoside B	M-H, M+Cl, M+K-2H, M+FA-H, 2M-H	C_21_H_32_O_12_	–	–3.9779	475.1802	475.1816	387.1263, 341.1218, 235.9249
12	3.88_480.0892n	Pyrone glycoside	Gossypetin-8-C-glucoside	M-H, 2M-H	C_21_H_20_O_13_	290, 347	–2.5323	479.0819	479.0819	303.0514, 167.0327
13	3.92_593.1501m/z	Flavonoid	Apigenin-6,8-digalactoside	M-H	C_27_H_30_O_15_	322	–1.8072	593.1501	593.1507	473.1048, 353.0662
14	4.11_449.1075m/z	Flavonoid	(*cis*)-5,7,2′-Trihydroxyflavanonol-3*-O*-β-D-glucopyranoside	M-H	C_21_H_22_O_11_	282	–3.179	449.1075	449.1084	287.0552
15	4.31_563.1398m/z	Flavonoid	Apigenin-6-C-glucoside 8-C-arabinoside	M-H	C_26_H_28_O_14_	271, 333	–1.4863	563.1398	563.1401	473.1092, 353.0658
16	4.50_449.1078m/z	Flavonoid	Flavanomarein	M-H	C_21_H_22_O_11_	282, 333	–2.5914	449.1078	449.1084	287.0558, 269.0471
17	4.50_463.0880m/z	Flavonoid	Isocarthamidin-7*-O*-D*-*glucuronide*	M-H	C_21_H_20_O_12_	246, 283	–0.4871	463.088	463.0877	285.0387
18	4.80_450.1150n	Dihydroflavones	Isookanin-7*-O*-glucoside	M-H2O-H, M+Na-2H	C_21_H_22_O_11_	289, 334	–2.6806	431.0972	431.0978	311.0541, 174.9557
19	4.84_464.0939n	Dihydroflavones	Carthamidin-7*-O*-D*-*glucuronide*	M+Na-2H, 2M-H, 2M+Hac-H, M-H	C_21_H_20_O_12_	246, 283, 347	–3.4338	463.0884	463.0877	287.0531
20	4.87_244.0658n		Unknown							
21	5.09_432.1076n	Flavonol	Kaempferol-7*-O*-rhamnoside	M-H2O-H, M-H	C_21_H_20_O_10_	282, 333	4.4983	431.0973	431.0978	285.0401
22	5.09_462.0788n	Flavonoid	Scutellarin*	M-H, 2M-H	C_21_H_18_O_12_	280, 331	–2.1404	461.0716	461.072	283.0239
23	5.13_285.0393m/z	Flavone	5,7,2′,5′-Tetrahydroxyflavone	M-H	C_15_H_10_O_6_	273	–4.0318	285.0393	285.0399	271.0351, 243.0292, 227.0345
24	5.26_581.1913m/z	Flavononol	Amoenin A	M-H	C_27_H_34_O_14_	287	6.4136	581.1913	581.1871	551.1733, 433.1083, 285.0387, 174.9557
25	5.33_447.0965m/z	Flavonoid	Luteolin-7*-O*-D*-*glucopyranside*	M-H	C_21_H_20_O_11_	282, 347	7.2842	447.0965	447.0928	271.0615
26	5.33_478.1097n	Flavonoid	5-(5,7-Dihydroxy-3-methoxy-4*-*oxo-4H-chromen-2-yl)-2-hydroxyphenyl β-D*-*glucopyranoside	M-H, M+Na-2H	C_22_H_22_O_12_	285, 334	–2.9882	477.1024	477.1033	301.0693, 271.0615, 174.9557
27	5.36_547.1452m/z	Flavonoid	Chrysin 6-C*-alpha*-L-arabinopyranoside-8-C-glucoside	M-H	C_26_H_30_O_14_	272, 319	–0.8791	547.1452	547.1452	427.1052, 367.0847, 337.0724
28	5.43_310.1041n		Unknown							
29	5.49_638.2191n	Flavonoid	Leucosceptoside A	M-H, M+K-2H	C_30_H_38_O_15_	275, 325	–3.0709	637.2118	637.2133	561.1579, 437.0855
30	5.55_415.1022m/z	Flavonoid	Chrysin-8-C-D-glucopyranside	M-H	C_21_H_20_O_9_	279, 329	–3.0133	415.1022	415.1029	295.0581, 267.0623
31	5.59_419.1329m/z	Others	2,3,8,9,10-Pentamethoxy-6a,11a-Dihydro-6H-[1] benzofuro[3,2-c]chromene	M+FA-H	C_20_H_22_O_7_	302	–5.0173	419.1329	419.1342	285.0395
32	5.62_462.0786n	Flavonoid	Kaempferol-3-glucuronide	M-H, M+Na-2H	C_21_H_18_O_12_	288, 322	–2.6553	461.0713	461.072	285.0387
33	5.65_432.1050n	Flavonoid	Apigenin-7-*O*-glucoside*	M-H, 2M-H	C_21_H_20_O_10_	280, 335	–1.4329	5.6545	431.0978	367.0850, 309.0404
34	5.69_288.0617n	Flavonoid	(2*S*) 5,7,2′6′-Tetrahydroxyflavanone	M-H, M+Na-2H, 2M-H, 2M+Hac-H	C_15_H_12_O_6_	288	–5.7107	287.0545	287.0556	125.0236
35	5.69_478.1095n	Flavonoid	5,7,2′-Trihydroxy-6-methoxyflavanone-7*-O-*β-D*-*glucuronoide	M-H, M+Na-2H, 2M-H	C_22_H_22_O_12_	285, 347	–3.3199	477.1023	477.1033	301.0706
36	5.69_547.1441m/		Unknown							
37	6.11_446.0844n	Flavonoid	Norwogonin-7*-O*-D*-*glucuronopyranoside	M-H, M+Na-2H	C_21_H_18_O_11_	288	–1.2162	445.0771	445.0771	291.0272
38	6.15_522.1367n	Flavonoid	(2*S*)5,2-Dihydroxy-7,8,6′-trimethoxyflavanone-2*-O*-D*-*glucuronide	M-H, M+Na-2H	C_24_H_26_O_13_	279, 333	–1.2292	543.0775	543.1115	413.0862, 287.0537
39	6.25_302.0775n	Others	Hematoxylin	M-H, 2M-H	C_16_H_14_O_6_	293	–5.2188	301.0701	301.0712	299.0553, 205.0492, 119.0491
40	6.25_475.0875m/z	Flavonoid	Scutevulin 2′*-O-*β-D*-*glucuronopyranoside	M-H	C_22_H_20_O_12_	272, 324	–1.3927	475.0875	475.0877	321.0374, 299.0553
41	6.39_652.2351n	Others	[5-Hydroxy-6-[2-(4-hydroxy-3-methoxyphenyl) ethoxy]-2-(hydroxymethyl)-4-(3,4,5-trihydroxy-6-methyloxan-2-yl) oxyoxan-3-yl] (E)-3-(4-hydroxy-3-methoxyphenyl) prop-2-enoate	M-H, M+Cl, M+FA-H, M-H2O-H	C_31_H_40_O_15_	288	–2.5089	651.2278	651.2289	475.1784, 175.0397
42	6.42_448.0991n	Flavonoid	Luteolin-4′*-O*-glucoside	M-H, M+Na-2H, M+K-2H, 2M+Hac-H, 2M-H	C_21_H_20_O_11_	282	–3.177	447.0919	447.0928	271.0615
43	6.54_467.0583m/z	Flavonoid	Baicalin*	M+Na-2H	C_21_H_18_O_11_	277, 314	–2.7721	467.0583	467.059	239.0354, 269.0431
44	6.64_286.0465n	Flavonoid	Scutellarein*	M-H, M+K-2H, 3M-H	C_15_H_10_O_6_	281, 322	–4.4541	285.0399	285.0399	117.0346
45	6.68_415.1024m/z	Flavonoid	Puerarin	M-H	C_21_H_20_O_9_	275, 347	–2.4492	415.1024	415.1029	325.0722, 295.0615, 267.0652
46	6.71_433.0766m/z	Flavonoid	Guajavarin	M-H	C_20_H_18_O_11_	273, 332	–2.3514	433.0766	433.0771	301.0353
47	6.78_462.0795n	Flavonoid	Isoscutellarein 8-glucuronide*	M-H, M+Na-2H, 2M-H	C_21_H_18_O_12_	288, 333	–0.8026	461.0721	461.072	285.0387, 174.9557
48	7.00_359.0763m/z	Flavonoid	5,6,2′-Trihydroxy-7,8,6′-trimethoxyflavone	M-H	C_18_H_16_O_8_	279	–2.6723	359.0763	359.0767	299.0551
49	7.10_452.2213n	Diterpenes	Scutebata J	M-H, M+Cl, M+FA-H	C_27_H_32_O_6_	–	3.227	497.2271	497.2175	371.1123
50	7.24_430.0906n	Flavonoid	Chrysin-7*-O*-D*-*glucuronopyranoside*	2M-H, 2M+FA-H, M-H, M+FA-H	C_21_H_18_O_10_	271, 309	1.4184	429.0829	429.0822	253.0500, 174.9557
51	7.37_857.1339m/z	Flavonoid	Luteolin*	3M-H	C_15_H_10_O_6_	256, 348	–2.0835	857.1339	857.1354	285.0406
52	7.46_467.2127m/z	Diterpenes	Scutebarbatine H	M-H	C_27_H_32_O_7_	–	11.0066	467.2127	467.2070	–
53	7.46_475.0900m/z	Flavonoid	Hispidulin-7*-O*-D*-*glucuronide	M-H	C_22_H_20_O_12_	273, 313	3.8674	475.09	475.0877	342.0745, 314.0643, 283.0606
54	7.56_230.0500n		Unknown							
55	7.56_283.0597m/z	Flavonoid	Acacetin	M-H	C_16_H_12_O_5_	–	–5.1247	283.0597	283.0607	268.0370, 163.0025
56	7.56_503.2461m/z	Diterpenes	Scutolide C	M-H2O-H	C_31_H_38_O_7_	–	4.1236	503.246	503.2434	462.2048, 225.0558
57	7.60_459.0952m/z	Flavonoid	Wogonoside*	M-H	C_22_H_20_O_11_	276	–1.3095	459.0952	459.0928	283.0617
58	7.83_467.0581m/z	Flavonoid	Apigenin-7*-O-*glucuronide	M+Na-2H	C_21_H_18_O_11_	267, 315	–3.257	467.0581	467.059	445.0802, 313.0707
59	8.12_270.0519n	Flavonoid	Apigenin*	M-H, 2M-H	C_15_H_10_O_5_	267, 339	–3.5697	269.0445	269.045	227.0343, 143.0473, 117.0339,
60	8.19_300.0620n	Flavonoid	5,7,2′-Trihydroxy-6′-methoxyflavone	M-H, M+K-2H	C_16_H_12_O_6_	281	–4.7514	299.0547	299.0556	269.0447, 151.0036, 143.0473, 117.0339
61	8.26_270.0888n	Flavonoid	Alpinetin*	M-H, 2M-H	C_16_H_14_O_4_	286	–1.4766		269.0814	227.0776, 184.0518
62	8.29_493.2254m/z	Diterpenes	Scutolide E	M-H	C_29_H_34_O_7_	–	–4.5466	493.2254	493.2227	447.2200
63	8.52_270.0528n	Flavonoid	Baicalein*	M-H, M+Na-2H, M+FA-H, 2M-H, 2M+Hac-H, M-H2O-H	C_15_H_10_O_5_	275, 324	0.0951	269.0455	269.045	239.0337, 195.0444
64	8.52_285.0388m/z	Flavonoid	3,6,3′,4′-Tetrahydroxyflavone	M-H	C_15_H_10_O_6_	274, 322	–5.833	285.0388	285.0399	239.0342, 211.0396, 195.0444
65	8.52_343.0809m/z	Flavonoid	Nevadensin	M-H	C_18_H_16_O_7_	272, 343	–4.1316	343.0809	343.0818	313.0352, 269.0456, 232.0572, 141.0496
66	8.55_299.0545m/z	Flavonoid	Scutellarein 4′-methyl ether	M-H	C_16_H_12_O_6_	280	–5.3997	299.0545	299.0556	269.0450
67	8.94_299.0550m/z	Flavonoid	5,7,2′-Trihydroxy-6′-methoxyflavone	M-H	C_16_H_12_O_6_	270, 335	–3.8571	299.055	299.0556	284.032
68	8.98_374.1003n	Flavonoid	Skullcapflavone II	M-H, M+Cl, M+K-2H, 2M-H	C_19_H_18_O_8_	281	0.3282	373.093	373.0924	373.093, 358.0685, 328.0220
69	9.31_255.0657m/z	Flavonoid	Pinocembrin*	M-H	C_15_H_12_O_4_	289, 348	–2.2057	255.0657	255.0658	169.0648
70	9.38_283.0618m/z	Flavonoid	Wogonin*	M-H	C_16_H_12_O_5_	276	–1.3095	283.0617	283.0618	163.0036
71	9.71_254.0586n	Flavonoid	Chrysin*	M-H, M+K-2H, M+Cl, 2M-H, 3M-H	C_15_H_10_O_4_	268, 313	2.5939	254.0586	254.0579	209.0603, 143.0502, 107.0122
72	9.77_283.0604m/z	Flavonoid	Oroxylin A*	M-H	C_16_H_12_O_5_	272, 320	–2.6747	283.0604	283.066	239.0354, 165.9893, 109.9997
73	10.06_538.0890n	Flavonoid	8,8′-Bibaiaclein	M-H, M+K-2H	C_30_H_18_O_10_	277	–1.9444	537.0817	537.0822	391.0448, 373.0354
74	10.17_581.2945m/z	Diterpenes	Scutefolide F	M+FA-H	C_29_H_44_O_9_	–	–4.2526	581.2945	581.2962	535.2905
75	10.23_343.0813m/z	Flavonoid	5,2′-Dihydroxy-6,7,8-trimethoxyflavone	M-H	C_18_H_16_O_7_	–	–2.8737	343.0813	343.0818	328.0576
76	10.46_459.2012m/z	Diterpenes	Scutegalerin E	M+Na-2H	C_23_H_34_O_8_	–	2.7113	459.2012	459.1995	365.1947
77	10.76_406.3089n	Diterpenes	5-[1,2,4a-Trimethyl-5-(3-methylbutanoyloxymethyl)-2,3,4,7,8,8a-hexahydronaphthalen-1-yl]-3-methylpentanoic acid	M+FA-H, 2M+FA-H, M+Cl	C_25_H_42_O_4_	–	1.4408	451.3071	451.306	405.1945, 117.055
78	10.80_405.1919m/z	Diterpenes	Scutebarbolide C	M-H2O-H	C_22_H_32_O_8_	–	–0.0388	405.1919	405.1913	311.1657, 301.1785
79	11.19_329.1746m/z	Diterpenes	Barbatin C	M-H2O-H	C_20_H_28_O_5_	–	–3.4979	329.1746	329.1753	329.1744, 285.1492, 139.1102
80	11.55_492.1656n	Diterpenes	Scutellarioside I	M-H, M+Na-2H, M-H2O-H	C_24_H_28_O_11_	–	4.8679	491.1583	491.1554	513.1390, 491.1558, 445.1524
81	12.99_476.3382n		Unknown							

*Identifications were confirmed by comparing t_R_ and MS spectra to standard compounds.

**FIGURE 3:**
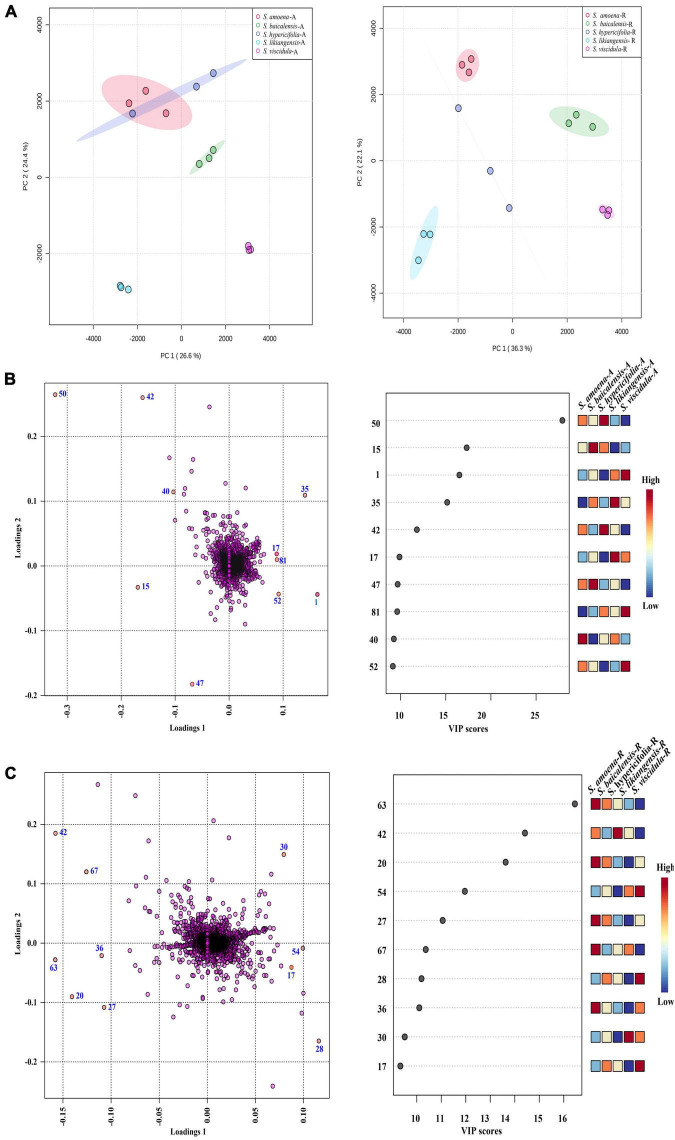
The multivariate analysis of the metabolite data was derived from five *Scutellaria* species. **(A)** PCA score map of the different parts of *Scutellaria* species (-A: aerial parts, -R: roots). **(B)** The loading plot and variable importance in the projection of the PLS-DA model for the aerial parts of *Scutellaria* species. **(C)** The loading plot and variable importance in the projection of the PLS-DA model for the roots of *Scutellaria* species. (The colored boxes on the right indicate the relative concentrations of the corresponding metabolite in each group under study; the number of the metabolites corresponds to Table 1).

Principal component analysis was first applied to analyze the chemical diversity of different parts among the tested *Scutellaria* species (Figure 3A). In this model, differences in the chemical composition of the five *Scutellaria* species were observed. The three substitute species other than *S. likiangensis* (*S. amoena, S. hypericifolia*, and *S. viscidula*) were similar in distance from *S. baicalensis* for both the aerial parts and roots, but *S. likiangensis* was more distant. In addition, the distance between *S. hypericifolia* and *S. amoena* was shorter for aerial parts.

Furthermore, partial least squares discriminant analysis (PLS-DA) was used to better describe the different chemical metabolic profiles of *S. baicalensis* and its four substitute species in different parts (Figure 3). The variable importance in the project (VIP) value reflects the influence of metabolites on classification. Therefore, metabolites with VIP values ranking in the top 10 were selected as potential markers to distinguish species (Figures 3B,C). In the aerial parts, the PLS-DA loading plot revealed that the separation of the species was mainly caused by pyrone glycoside, flavonoids, and diterpenes. Moreover, the compound scutevulin-2′-*O*-β-D-glucuronopyranoside (**40**) showed the highest relative content in *S. amoena*, the compounds apigenin-6-C-glucoside-8-C-arabinoside (**15**) and isoscutellarein-8-glucuronide (**47**) were more abundant in *S. baicalensis*, and the compounds luteolin-4′-*O*-glucoside (**42**) and chrysin-7-*O*-D-glucuronopyranoside (**50**) displayed the highest content in *S. hypericifolia*. The compounds isocarthamidin-7-*O*-D-glucuronide (**17**) and 5,7,2′-trihydroxy-6-methoxyflavanone-7-*O*-β-D-glucuronide (**35**) were more abundant in *S. likiangensis*, and the compounds scusalvioside A (**1**), scutebarbatine H (**52**) and unknown (**81**) demonstrated relatively high contents in *S. viscidula*. In the PLS-DA plot for the roots, *S. amoena* showed relatively high concentrations of baicalein (**63**), unknown (**20**), chrysin 6-C-alpha-L-arabinopyranoside-8-C-glucoside (**27**), 5,7,2′-trihydroxy-6′-methoxyflavone (**67**), and unknown (**36**); *S. hypericifolia* showed the highest concentration of luteolin-4′-*O*-glucoside (**42**); *S. likiangensis* showed the highest concentration of chrysin-8-C-D-glucopyranoside (**30**); and *S. viscidula* displayed the highest concentrations of isocarthamidin-7-*O*-D-glucuronide (**17**) and unknown (**28, 54**). The unknown metabolites **20**, **28, 36, 54,** and **81** were not annotated because their m/z and possible molecular formulas did not match any of the previously reported compounds.

### Quantitative determination of the 15 assessed chemical components in the *Scutellaria* species

**FIGURE 4:**
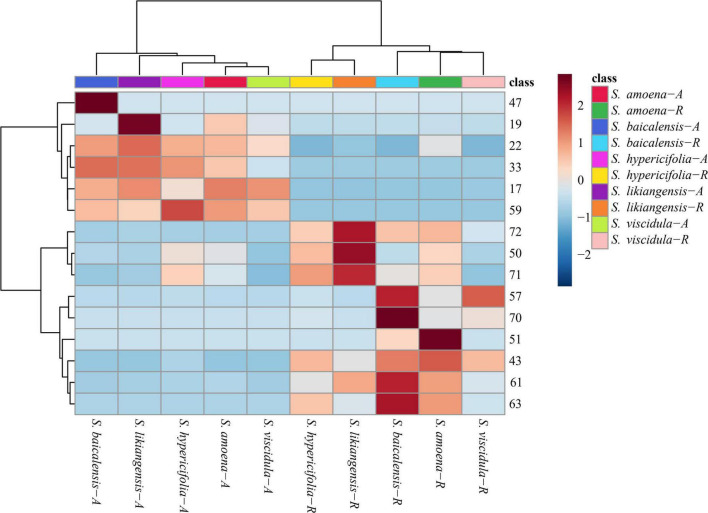
Clustering heatmap of 15 compounds in different parts of five *Scutellaria* species (the number of analytes corresponds to Table 1; -A: aerial parts, -R: roots).

The HPLC analytical method described in our previous reports (Shen et al., 2019) was used to simultaneously quantify 15 compounds in every sample. The analytical results are shown in Figure 4 and Supplementary Table S3. In the aerial parts of five *Scutellaria* species, the contents of isocarthamidin-7-*O*-D-glucuronide (**17**), carthamidin-7-*O*-D-glucuronide (**19**), scutellarin (**22**), apigenin-7-*O*-β-D-glucopyranoside (**33**), isoscutellarein-8-glucuronide (**47**), and apigenin (**59**) were higher than those in roots. Of these, compounds **17, 19, and 22** were higher in the aboveground parts of the five species of *Scutellaria*. In addition, the contents of some compounds were consistent with the relative abundances of compounds identified in the PLS-DA pattern. In the aerial parts, the concentration of isocarthamidin-7-*O*-D-glucuronide (**17**) was significantly lower in *S. hypericifolia* (53.6195 ± 18.9497 mg/g) and that of isoscutellarein-8-glucuronide (**47**) was significantly higher in *S. baicalensis* (5.1336 ± 1.6451 mg/g). In addition, carthamidin-7-*O*-D-glucuronide (**19,** 120.5610 ± 5.0985 mg/g) and scutellarin (**22,** 26.5083 ± 3.4493 mg/g) displayed the highest contents in *S. likiangensis*. Apigenin-7-*O*-β-D-glucopyranoside (**33**) showed the lowest content (1.6602 ± 0.2706 mg/g) in *S. viscidula*. Apigenin (**59**) displayed a relatively high content in *S. hypericifolia* (0.0802 ± 0.0175 mg/g).

Nine compounds, baicalin (**43**), chrysin-7-*O*-D-glucuronopyranoside (**50**), luteolin (**51**), wogonoside (**57**), alpinetin (**61**), baicalein (**63**), wogonin (**70**), chrysin (**71**), and oroxylin A (**72**), were more abundant in the roots of these five *Scutellaria* species. In addition, the contents of chrysin-7-*O*-D-glucuronopyranoside (**50**), chrysin (**71**), and oroxylin A (**72**) in *S. likiangensis* were higher at 69.7029 ± 19.0448, 1.6112 ± 0.6629, and 2.7468 ± 0.6434 mg/g, respectively. Wogonoside (**57**), alpinetin (**61**), baicalein (**63**), and wogonin (**70**) were more abundant in *S. baicalensis*, with contents of 31.6445 ± 0.9172, 1.8570 ± 0.1488, 14.6276 ± 0.9966, and 0.0077 ± 0.0021 mg/g, respectively. The compounds baicalin (**43**) and luteolin (**51**) were more abundant in *S. amoena*. Furthermore, the dried root of *S. baicalensis* (Scutellariae Radix) is a typical Chinese materia medica. According to the Chinese pharmacopoeia (2020 edition), the content of baicalin in Scutellariae Radix should not be less than 9.0%. In this study, in contrast to the content in *S. likiangensis* (50.1253 ± 14.5487 mg/g), the contents of baicalin (**43**) were higher than 9.0% in the roots of *S. amoena* (148.8557 ± 47.1488 mg/g), *S. baicalensis* (130.6066 ± 9.3707 mg/g), *S. hypericifolia* (96.1738 ± 28.3923 mg/g), and *S. viscidula* (93.5047 ± 8.3265 mg/g) (Supplementary Table S3).

## Discussion

In this study, the complete cp genomes of four species of *Scutellaria* (*S. amoena*, *S. hypericifolia*, *S. likiangensis*, *S. viscidula*) were assembled for the first time, and comparative and phylogenetic analyses with other related *Scutellaria* species were also performed (Lee and Kim, 2019; Liu et al., 2020; Zhao et al., 2020). The four *Scutellaria* cp genomes have high similarities in genome structure and size, gene number, and the distribution of repeat sequences. Furthermore, one high-frequency sequence variation intergenic region (*petA-psbJ*) was found among the above four *Scutellaria* species and *S. baicalensis*. The fragment *petA-psbJ* has been used in previous phylogenetic studies (Latvis et al., 2017) but not in Lamiaceae. *petA-psbJ* may represent a potential barcode specific to these *Scutellaria* species that are currently available. In addition, the phenomena of expansion and contraction of the IR region of the among the species of *Scutellaria* have been observed. Changes in the boundaries of LSCs and IRs are relatively common in plants (Li et al., 2018; Yan et al., 2019; Zhao et al., 2020). Similar to the findings of Zhao et al. (2020), the changes in the LSC region were larger, which indicates that divergence in LSC length leads to variation in the size of the cp genomes based on IR contraction or expansion. During the evolution of cp genomes, differences in the four IR boundaries among species have been frequently observed, leading to further variation in cp genome size. Thus, although IR regions are the most conserved regions in the cp genome sequence, they have been used to explain differences in size among cp genomes due to contraction and expansion at their boundaries (Liu et al., 2019; Xue et al., 2019). In addition, the location of the IR boundary can be used to study phylogeny; in closely related species, small expansions or contractions tend to have similar endpoints (Cui et al., 2019b).

Morphological polymorphisms make the classification of *Scutellaria* extremely difficult, and it is also challenging to define species, groups, and phylogenetic relationships within the genus. *Scutellaria* is a very isolated genus with unsatisfactory traditional divisions. Currently, the commonly used classification method in China is based on the *Flora of China* (1977) (Wu and Li, 1977). In this study, phylogenetic analyses showed that the cp-based phylogenetic tree and traditional plant classification of *Scutellaria* in China were incongruent. According to the *Flora of China* (1977), the species of *S. hypericifolia*, *S. baicalensis*, and *S. viscidula* are grouped, while in the cp-based phylogenetic tree, *S. hypericifolia* is clustered with *S. likiangensis* and *S. viscidula*. In addition, it was observed that *S. baicalensis* and its substitute species may be a special group in the genus *Scutellaria*.

Phylogenetically closely related species often share similar chemical characteristics and even similar clinical efficacy, which is the core idea of pharmacophylogeny (Gong et al., 2020; Hao and Xiao, 2020). The 17 species of genus *Scutellaria* involved in the cp genome phylogenetic tree are classified into two main branches, where the presence or absence of a well-developed root is a significant morphological difference between Subclade I and Subclade II. Subclade I can be divided into two clades, Subclade I_1_ and Subclade I_2_. Subclade I_1_ contains the species *S. altaica* and *S. przewalskii*, both of which are perennial subshrubs with developed woody rhizomes and no phytochemical studies. In Subclade I_2_, *S. kingiana* is a dwarf perennial herb and has not been chemically studied. The species of *S. amoena*, *S. baicalensis*, *S. hypericifolia*, *S. likiangensis*, and *S. viscidula* are clustered together and are all erect or ascending erect perennial herbs with fleshy rhizomes. As in previous reports of *S. baicalensis*, there are significant differences in the chemical composition between their aerial parts and roots (Shen et al., 2019), while both are reported to have various benefits for human health, such as anti-aging, anti-inflammation, and antitumor activities, which are mainly attributed to flavonoids (Gao et al., 2016; Zhang et al., 2017; Shen et al., 2019, 2020; Song et al., 2020). In this study, we further explored the chemical characteristics of different parts (aerial parts and roots) of these species. The four substitute medicinal species and *S. baicalensis* share similarity in chemical composition both in aerial parts and roots. It is reported that the 15 accessed chemical components were the active or main compounds in the species of *S. baicalensis*. Among these compounds, **19, 22, 33, 43, 47, 50, 51, 57,** and **59** were all detected in the aerial parts of the four substitute medicinal species for *S. baicalensis*. The compounds isocarthamidin-7-*O*-D-glucuronide (**17**), scutellarin (**22**), and apigenin (**59**) showed higher contents and various activities (Li et al., 2017; Wang and Ma, 2018; Oaarowski et al., 2019) in the aerial parts. Moreover, the content of scutellarin (**22**, 26.5083 ± 3.4493) was highest in the aerial parts of *S. likiangensis*, which could be a good choice as a natural source of scutellarin for making cerebrovascular and cardiovascular drugs (Breviscapin) (Wen et al., 2021). In particular, it has been reported that the pharmacological activities of Scutellariae Radix are closely related to flavones lacking 4’-OH groups on the B-rings (Zhao et al., 2018; Song et al., 2020; Sun et al., 2020), such as baicalin (**43**), wogonoside (**57**), baicalein (**63**), wogonin (**70**), chrysin (**71**), and oroxylin A (**72**). The roots of the four substitute species all contain these special compounds. The compounds in the roots of *S. amoena* were similar to those in the roots of *S. baicalensis*. In addition, according to the standard of the Chinese pharmacopoeia (baicalin > 9%), *S. likiangensis* could not be used as a substitute species for *S. baicalensis*. Subclade II consists of nine species: *S. calcarata, S. lateriflora, S. indica* var. *coccinea, S. insignis, S. mollifolia, S. orthocalyx, S. quadrilobulata, S. tsinyunensis*, and *S. scordifolia*, and all of them have short roots and are small plants. Among them, only *S. lateriflora* and *S. scordifolia* have had limited chemical studies. *S. lateriflora* contains both flavonoids and diterpenoids, and only flavonoids have been reported for *S. scordifolia*. Currently, flavonoids and diterpenes are thought to be the two main active constituent groups in the genus *Scutellaria*. The relationship of patterns of major chemical diversity to the known phylogeny of these *Scutellaria* species are still unclear due to the lack of adequate studies on phylogeny, chemical composition and constituent contents. In further studies, additional evidence is needed to confirm the relationship between the chemical diversity and the known phylogeny in *Scutellaria*.

The species *S. amoena*, *S. hypericifolia*, *S. likiangensis*, and *S. viscidula* have been traditionally used for a long time as substitutes for *S. baicalensis* in their places of origin. In recent years, this substitution has gradually decreased due to the expansion of areas of artificial cultivation of *S. baicalensis* and declines in the availability of wild resources. However, as these substitutes for *S. baicalensis* have been included in the local standards, long-term use habits are not applied differently. Considering the differences in geographical distribution, growth environment, and species genetics, our attention to the possible nuances in efficacy is still needed. Although the present study revealed that their primary chemical composition is similar, there are differences in the types of compounds and relative contents of the main active ingredients. Therefore, the similarities and differences in their pharmacological activities still need to be further investigated to ensure the validity and stability of the quality of the medicinal herbs.

## Conclusion

In this study, the complete cp genomes of four species of *Scutellaria* (*S. amoena, S. hypericifolia, S. likiangensis*, and *S. viscidula*) were sequenced by high-throughput sequencing. In addition, the complete cp genomes and chemical analysis of *S. hypericifolia, S. likiangensis*, and *S. viscidula* are reported for the first time. Through comparison, we found that the cp genomes of these four species have similar structural characteristics and the typical four-part structure of other land plants. The nucleotide variation analysis showed that the mutational hot spot of *petA*-*psbL* could be used as an effective molecular marker for distinguishing *S. baicalensis* from its substitutes (*S. amoena* and *S. hypericifolia, S. likiangensis*, and *S. viscidula*). The phylogenetic analysis showed that the traditional morphological classification method in China is different from the cp-based phylogenetic tree, which suggests that the subdivision of the genus *Scutellaria* should be reconsidered. Additionally, the chemical distributions of four *Scutellaria* species determined using UPLC-QTOF-MS and HPLC analyses revealed that each *Scutellaria* plant exhibits a chemical metabolism similar to that of *S. baicalensis*. Further investigations are needed to reveal the links between the genetic and chemical relationships of *Scutellaria* species; however, information on these four complete cp genomes of *Scutellaria* will provide a valuable medicinal genetic resource for *Scutellaria* species authentication and phylogenetic analysis, and this comparative study will be a basis for further studies on the genus *Scutellaria.*

## Data availability statement

The data presented in this study are deposited in GenBank, accession numbers MW286269–MW286272.

## Author contributions

JS wrote the manuscript. CH and PX systemically revised the manuscript for important content. JS, YW, and PL helped to complete the data analysis. JS, YL, and KY completed the figures and tables. QW helped to collect and identify the samples. HY and QW reviewed and edited the original manuscript. CH proposed the conception. JS designed the structure of the manuscript. All authors read and approved the final manuscript.

## Footnotes

1
https://chlorobox.mpimp-golm.mpg.de/geseq.html

2
http://ogdraw.mpimp-golm.mpg.de/

3
http://pgrc.ipk-gatersleben.de/misa

4
http://tandem.bu.edu/trf/trf.html

5
http://genome.lbl.gov/vista/mvista/submit.shtml

6
https://mafft.cbrc.jp/alignment/server/index.html

7
https://irscope.shinyapps.io/irapp/

8
https://www.metaboanalyst.ca/
