# Supplementary material for: Pharmacophylogenetic study of Scutellaria baicalensis and its substitute medicinal species based on the chloroplast genomics, metabolomics, and active ingredient
Source: Front Plant Sci. 2022 Aug 17;13:951824. doi: 10.3389/fpls.2022.951824 (PMC9433114; doi:10.3389/fpls.2022.951824)
Supplement: Supplementary file 1 [file Data_Sheet_1.docx]

**Table S1, Comparisons among the cp genome characteristics of *S. amoena*, *S. hypericifolia*，*S. likiangensis,* and *S. viscidula***

|  |  | *S. amoena* | *S. hypericifolia* | *S. likiangensis* | *S. viscidula* | |
| --- | --- | --- | --- | --- | --- | --- |
| **Size (bp)** | Total | 151655 | 151574 | 151642 | | 151816 |
|  | LSC | 83823 | 83742 | 83810 | | 83995 |
|  | SSC | 17326 | 17326 | 17326 | | 17321 |
|  | IR | 25253 | 25253 | 25253 | | 25265 |
| **GC (%)** | IR | 43.64 | 43.64 | 43.64 | | 43.61 |
|  | LSC | 36.34 | 36.36 | 36.35 | | 36.34 |
|  | SSC | 32.71 | 32.72 | 32.71 | | 32.68 |
|  | Total | 38.36 | 38.37 | 38.36 | | 38.34 |
| **Genes number** |  | 113 | 113 | 113 | | 113 |
| **Protein coding number** |  | 80 | 80 | 80 | | 80 |
| **tRNA genes** |  | 29 | 29 | 29 | | 29 |
| **rRNA genes** |  | 4 | 4 | 4 | | 4 |

**Table S2 List of genes in the chloroplast genome of *Scutellaria***

| Category | Type of genes | Name of genes | Num. |
| --- | --- | --- | --- |
| Photosynthesis | Photosystem I | *psaA，psaB，psaC**，psaI，psaJ* | 5 |
|  | Photosystem II | *psbA, psbB, psbC, psbD, psbE, psbF, psbH, psbI, psbJ, psbK, psbL, psbM, psbN, psbT, psbZ* | 15 |
|  | Cytochrome b/f complex | *petA, petB*, petD*, petG, petL, petN* | 6 |
|  | ATP synthase | *atpA, atpB, atpE, atpF*, atpH, atpI* | 6 |
|  | NADH dehydrogenase | *ndhA*，ndhB*(2X), ndhC，ndhD，ndhE，ndhF，ndhG，ndhH，ndhI，ndhJ，ndhK* | 12 |
|  | Rubisco large subunit | *rbcL* | 1 |
| Self-replication | RNA polymerase | *rpoA, rpoB, rpoC1*, rpoC2* | 4 |
|  | Ribosomal RNA genes | *rrn16 (2X), rrn23 (2X), rrn4.5 (2X), rrn5 (2X)* | 8 |
|  | Small ribosomal proteins (SSU) | *rps2, rps3, rps4, rps7 (2X), rps8, rps11, rps12* (3X), rps14, rps15, rps16*, rps18, rps19* | 15 |
|  | Ribosomal proteins (LSU) | *rpl2* (2X), rpl14, rpl16*, rpl20, rpl22, rpl23 (2X), rpl32, rpl33, rpl36* | 11 |
|  | Transfer RNAs | *trnN-GUU(2X), trnR-ACG(2X), trnA-UGC*(2X), trnI-GAU*(2X), trnV-GAC(2X),* *trnL-CAA(2X), trnI-CAU(2X), trnH-GUG, trnK-UUU*, trnQ-UUG, trnS-GCU, trnG-UCC*, trnR-UCU, trnC-GCA, trnD-GUC, trnY-GUA, trnT-GGU, trnS-UGA, trnG-GCC, trnfM-CAU, trnS-GGA, trnT-UGU, trnL-UAA*, trnF-GAA, trnV-UAC*，trnM-CAU, trnW-CCA, trnP-UGG, trnL-UAG* (6 contain one intron, 7 are duplicated in the IR region) | 36 |
| Other genes | Translation initiation factor | *infA* | 1 |
|  | Maturase K | *matK* | 1 |
|  | Protease | *clpP*** | 1 |
|  | Envelope membrane protein Subunit | *cemA* | 1 |
|  | Subunit of acetyl-CoA-carboxylase | *accD* | 1 |
|  | c-type cytochrome synthesis gene | *ccsA* | 1 |
|  | hypothetical chloroplast reading frames (*ycf*) | *ycf1, ycf2 (2X), ycf3**, ycf4, ycf15* *(2X)* | 7 |

*Gene with a single intron, **gene with two introns, (2X) duplicated gene.

**Table S3. Contents of 15 compounds in different parts of 5 *Scutellaria* species (mean ±SD, mg/g, n=3; the number of analytes corresponds to Table 1)**

|  |  | Aerial parts | | | | | Roots | | | | |
| --- | --- | --- | --- | --- | --- | --- | --- | --- | --- | --- | --- |
| NO. | Analyte | *S. amoena* | *S. baicalensis* | *S. hypericifolia* | *S. likiangensis* | *S. viscidula* | *S. amoena* | *S. baicalensis* | *S. hypericifolia* | *S. likiangensis* | *S. viscidula* |
| **17** | Isocarthamidin-7*-O*-D*-*glucuronide | 109.5242±44.6433 | 88.4285±20.6736 | 53.6195±18.9497 | 104.1892±29.0588 | 101.0172±15.8649 | 2.0752±1.3841 | 1.7670±0.4028 | 1.4507±0.9512 | 0.6077±0.1372 | 4.8490±0.2953 |
| **19** | Carthamidin-7*-O*-D*-*glucuronide | 63.3063± 38.6005 | 14.3421±7.4375 | 12.0599±8.2974 | 120.5610±5.0985 | 19.5860±3.5152 | 4.8115±0.7105 | 1.9458±2.0084 | - | 1.0175±1.0570 | - |
| **22** | Scutellarin | 18.8468±1.3838 | 21.8082±6.9441 | 19.7284±6.5141 | 26.5083±3.4493 | 14.4600±2.5044 | 10.4624±1.6735 | - | - | 2.5285±0.2970 | - |
| **33** | Apigenin-7-*O-β*-D-glucopyranside | 4.3784±0.4914 | 7.1074±2.5394 | 5.9757±4.4574 | 6.8663±5.9537 | 1.6602±0.2706 | - | - | - | - | - |
| **43** | Baicalin | 0.4685±0.2119 | 2.3463±0.6294 | 15.8353±13.7301 | 1.6741±0.6948 | 1.4532±0.3275 | 148.8557±47.1488 | 130.6066±9.3707 | 96.1738± 28.3923 | 50.1253±14.5487 | 93.5047±8.3265 |
| **47** | Isoscutellarein 8-glucuronide | - | 5.1336±1.6451 | - | - | - | - | - | - | - | - |
| **50** | Chrysin-7*-O*-D*-*glucuronopyranoside | 17.2239±10.7974 | 6.7973±5.1227 | 21.7433±3.6460 | 5.1095±1.0738 | 0.3711± 0.0474 | 26.7363±5.8101 | 8.9987±0.5956 | 33.1390±24.9495 | 69.7029±19.0448 | 5.3779±0.4370 |
| **51** | Luteolin | - | - | - | - | - | 0.1598±0.1444 | 0.0411±0.0711 | - | - | - |
| **57** | Wogonoside | 0.0753±0.0022 | 0.2154±0.1429 | 0.8423±1.2682 | 0.0012±0 | - | 5.6027±3.8493 | 31.6445±0.9172 | 1.9936±0.5260 | 0.2979±0.0174 | 25.3901±1.8782 |
| **59** | Apigenin | 0.2408±0.1501 | 0.1943±0.0960 | 0.3291±0.4592 | 0.1638±0.0670 | 0.0263 | - | - | - | - | - |
| **61** | Alpinetin | - | - | 0.0802±0.0175 | 0.0362±0 | 0.0019±0.0000 | 1.1420±0.8602 | 1.8570±0.1488 | 0.4605±0.2428 | 1.0636±0.1871 | 0.3423±0.0671 |
| **63** | Baicalein | - | - | - | - | - | 8.6013±5.7366 | 14.6276±0.9966 | 6.2434±0.4734 | 2.5310±0.5135 | 1.8815±0.4722 |
| **70** | Wogonin | - | - | - | - | - | 0.0009±0.0006 | 0.0077± 0.0021 | 0.0004± 0.0001 | - | 0.0013±0.0002 |
| **71** | Chrysin | 0.3909±0.1660 | 0.0754±0.0408 | 0.7395±0.6818 | 0.1146±0.0337 | **-** | 0.7564± 0.3436 | 0.5110±0.2386 | 1.0598±0.7812 | 1.6112±0.6629 | 0.0340±0.0223 |
| **72** | Oroxylin A | - | - | 0.0158±0.0274 | 0.0611±0.0247 | **-** | 1.3651±0.6414 | 1.2449±0.6940 | 1.1067±0.9195 | 2.7468±0.6434 | 0.4267±0.0358 |

—: means undetected compound

(C)


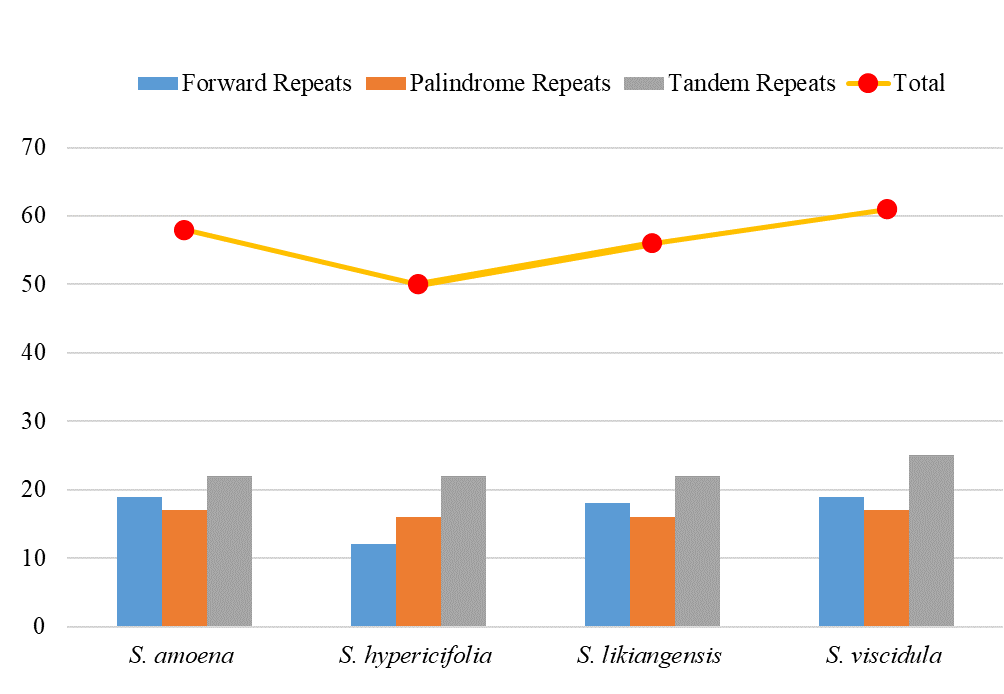


(A)

(B)


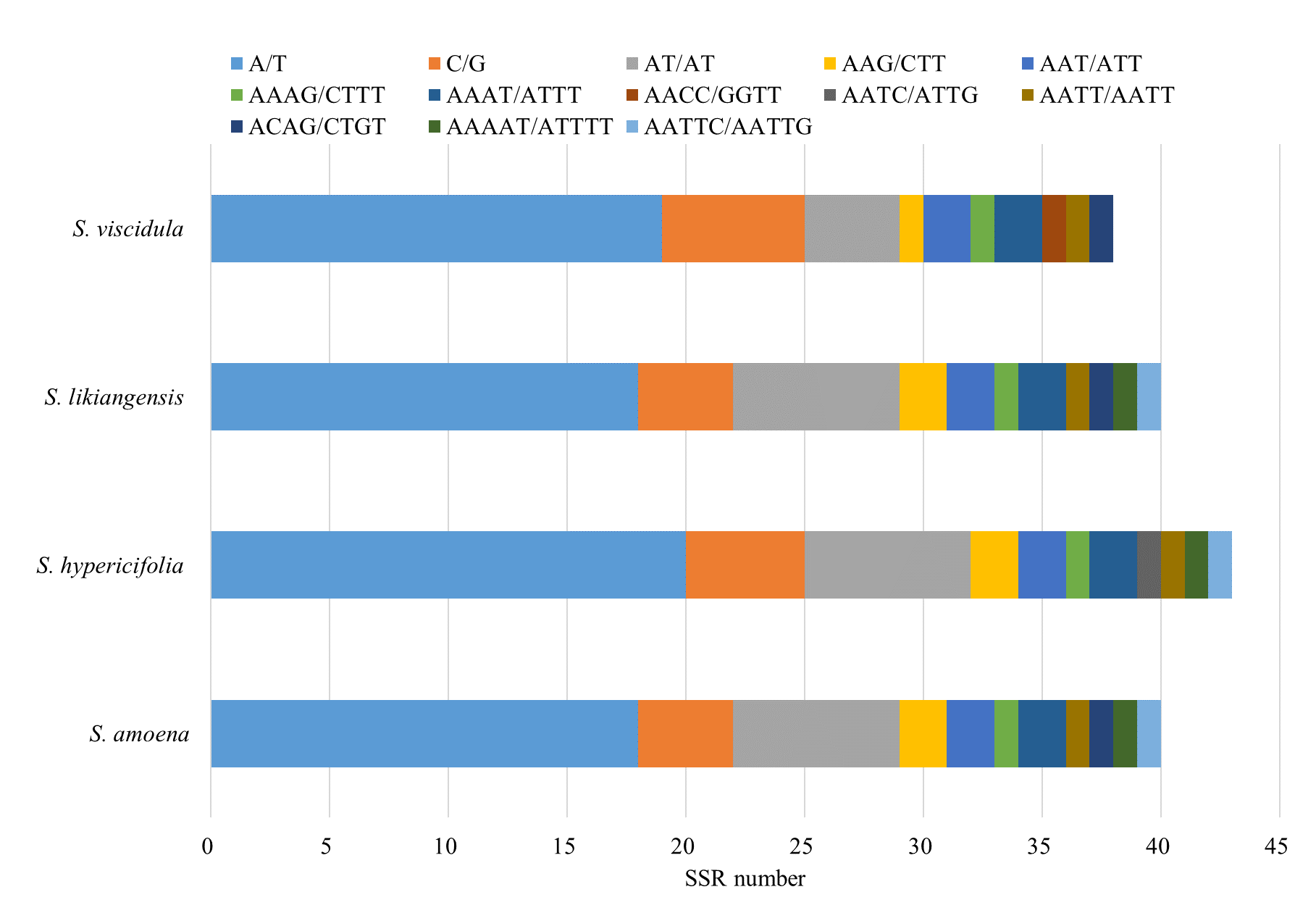

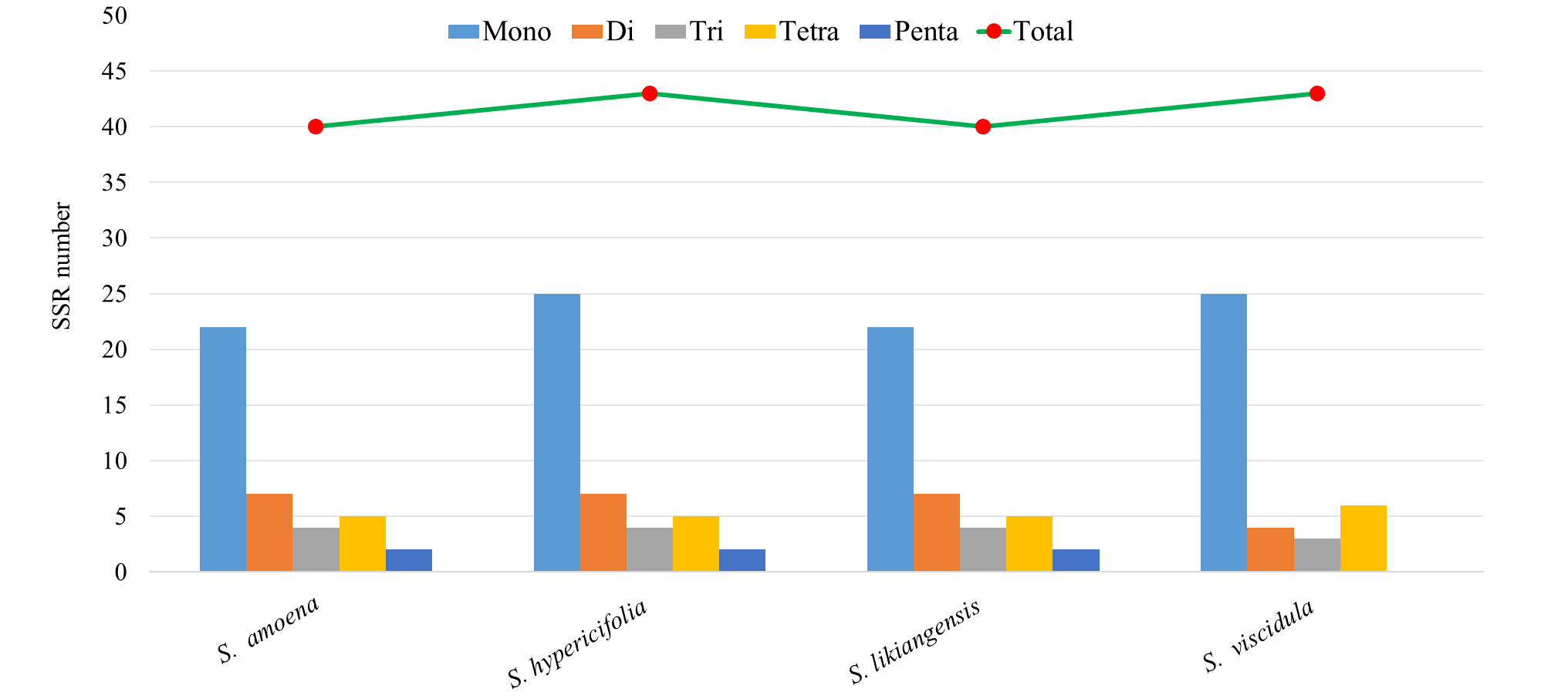


Figure S1. The number and type of SSR types and repeat types detected in each plastome of *S. amoena*, *S. hypericifolia, S. likiangensis,* and *S. viscidula*. (A) Frequency of SSR types, (B) frequency of classified SSR types (considering sequence complementarity), and (C) number of forward repeats, palindrome repeats, and tandem repeats.


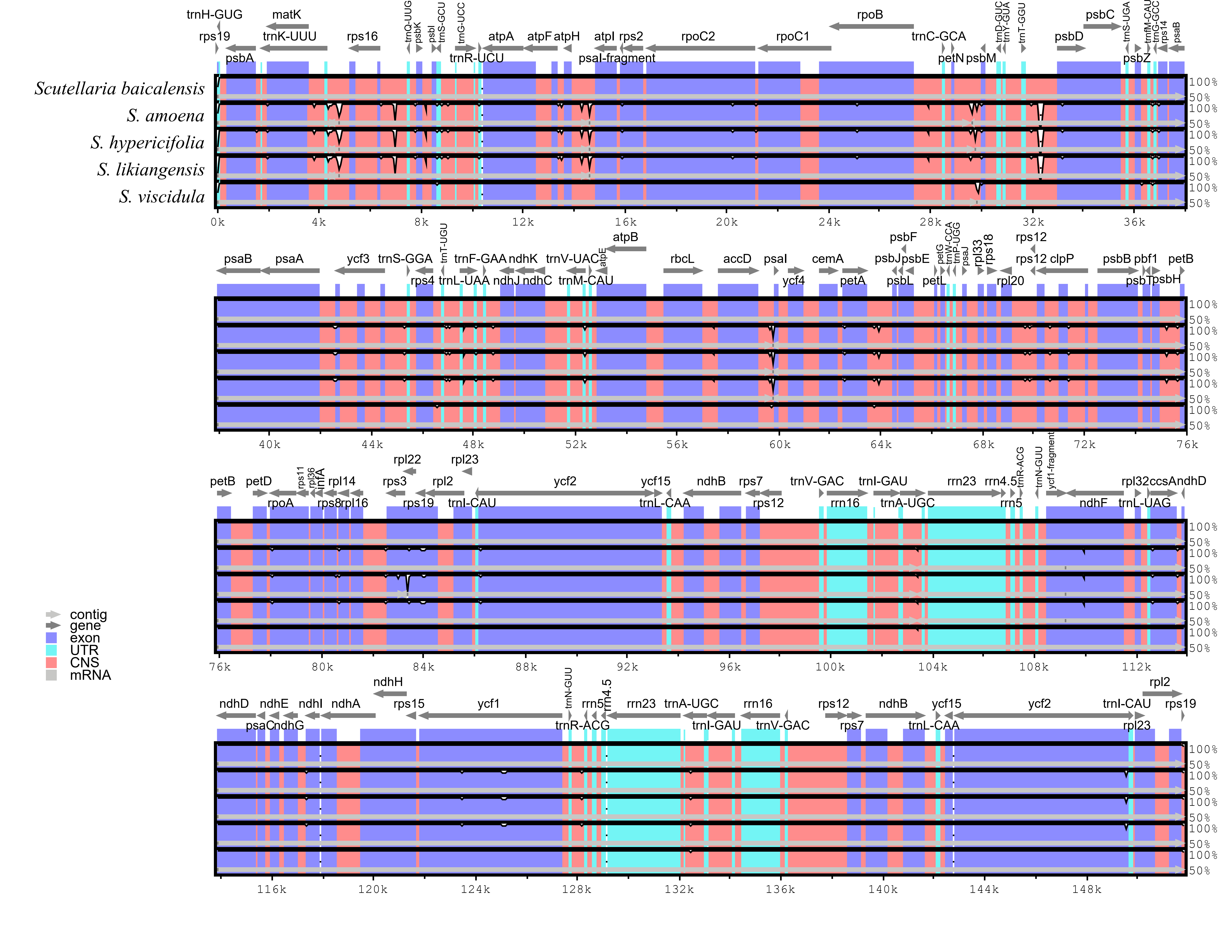


Figure S2 Sequence alignment of the whole plastomes of 5 taxa of *Scutellaria* using the LAGAN alignment algorithm in mVISTA, with *Scutellaria baicalensis* as the reference. Note：Grey arrows and thick black lines above the alignment indicate gene orientation; The Y-axis represents percent identity in the 50-100% range; Purple bars represent exons，blue bars represent UTRs，and pink bars represent non-coding sequences.


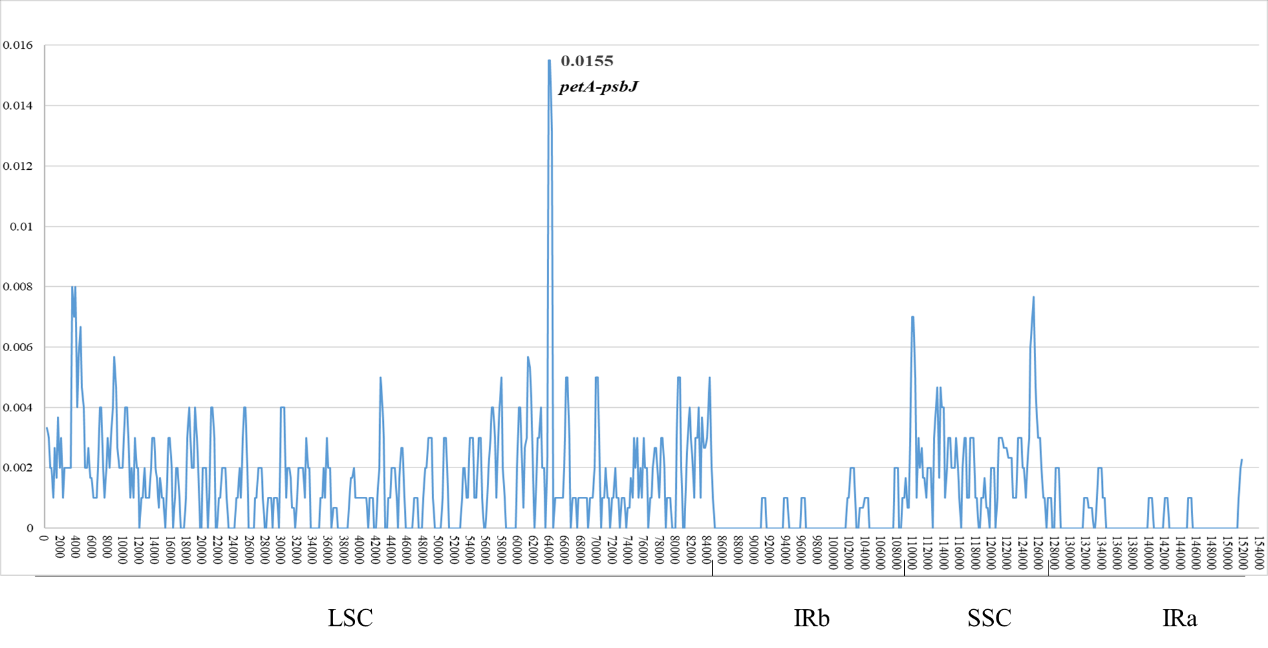


Figure S3 Sliding window analysis of the cp genomes of five *Scutellaria species* (*S. amoena*, *S. baicalensis, S. hypericifolia, S. likiangensis,* and *S. viscidula*). Window length: 800 bp; step size: 200 bp. X-axis: position of the midpoint of a window. Y-axis: nucleotide diversity of each window


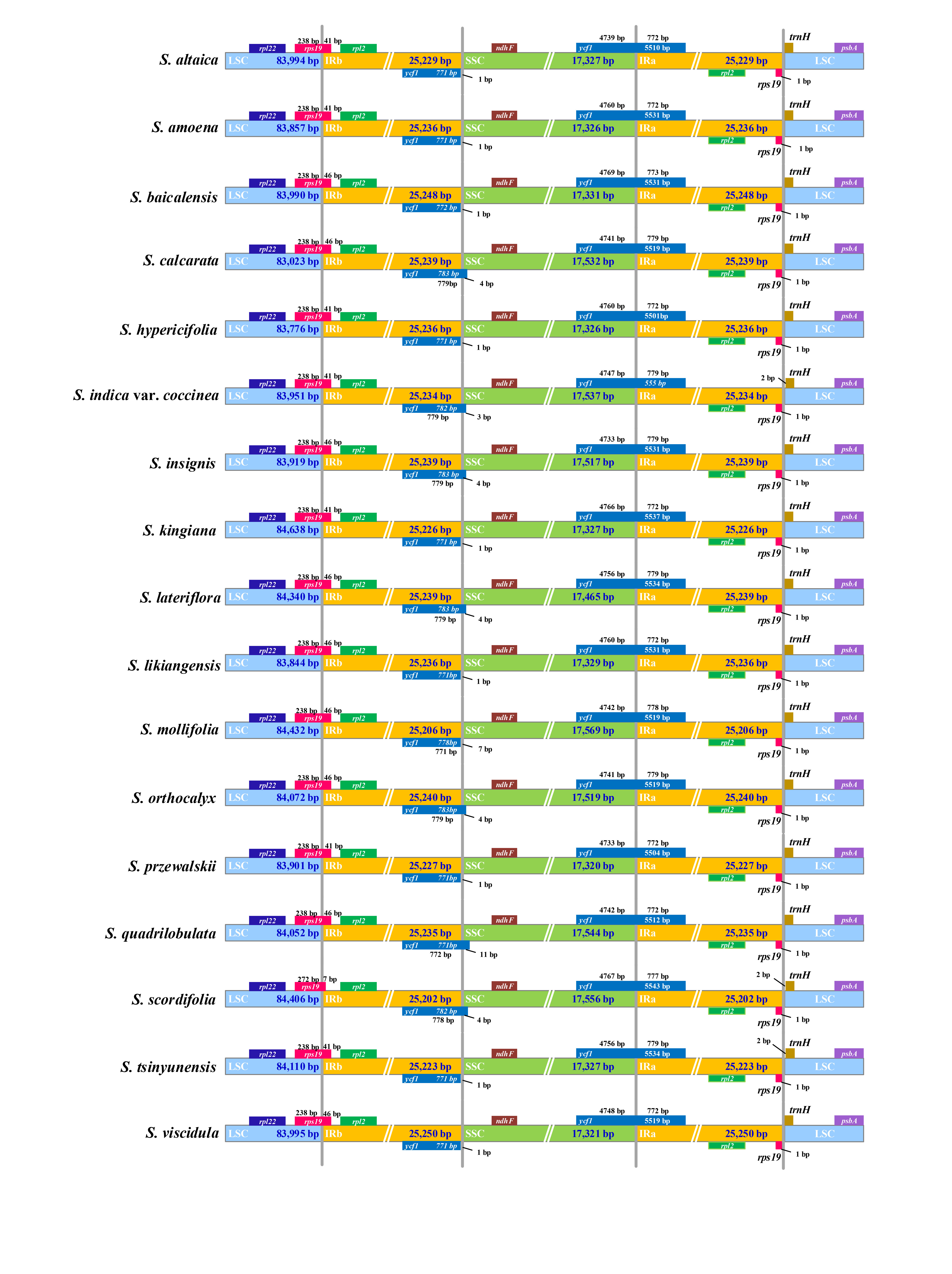


Figure S4. Comparison of LSC, IRb, SSC, and IRa border regions in 17 species of *Scutellaria*.


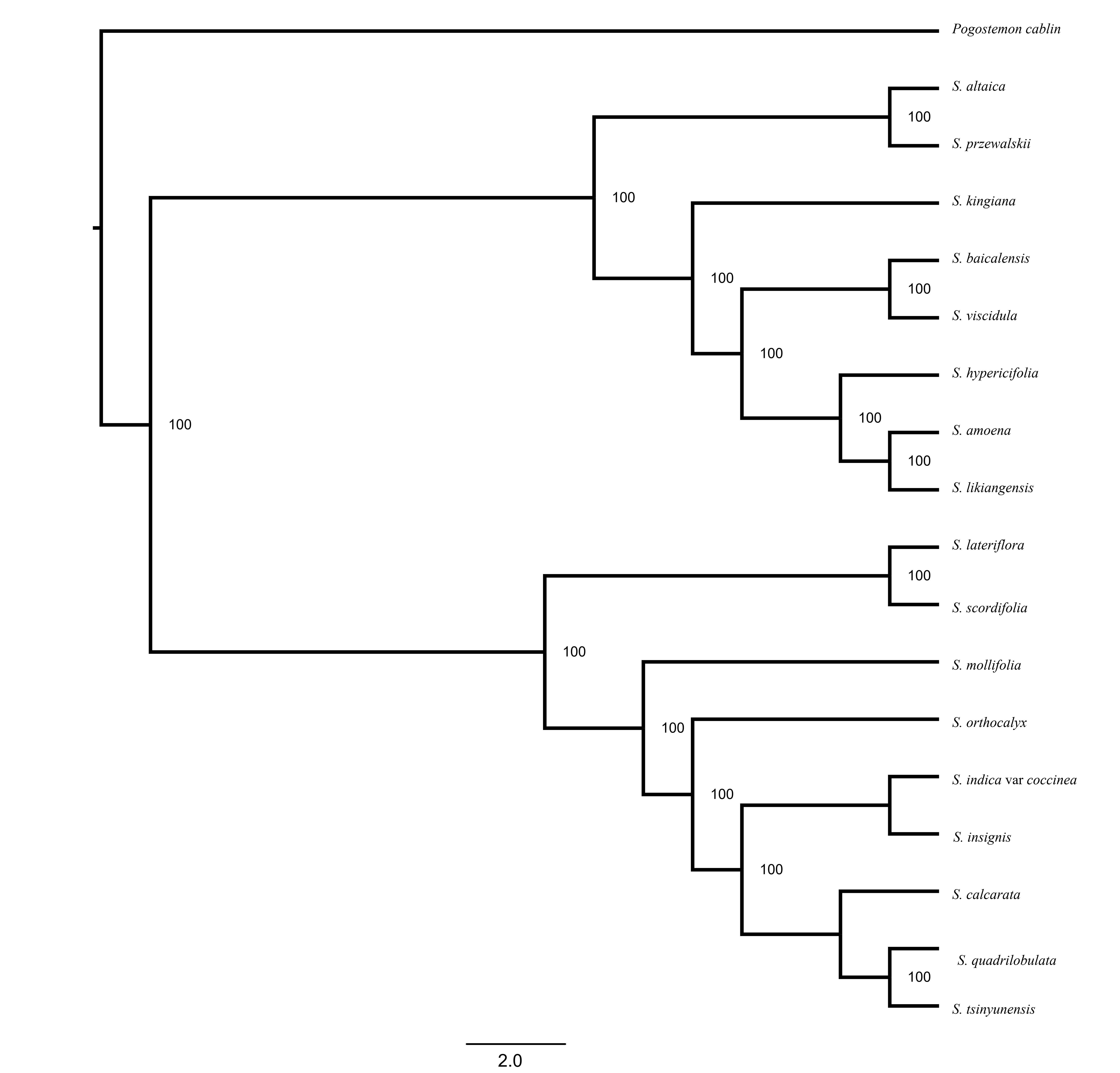


Figure S5 Phylogenetic tree inferred from Bayesian inference (BI) based on the whole chloroplast genomes. Numbers above the lines represent the Bayesian posterior probabilities.
